# Toward Optimal Care for Children With Congenital Unilateral Aural Atresia

**DOI:** 10.3389/fneur.2021.687070

**Published:** 2021-07-08

**Authors:** Filip Asp, Robert J. Stokroos, Martijn J. H. Agterberg

**Affiliations:** ^1^Scientific Center for Advanced Pediatric Audiology, Division of Ear, Nose, and Throat Diseases, Department of Clinical Science, Intervention and Technology Karolinska Institute, Stockholm, Sweden; ^2^Department of Otorhinolaryngology, Head and Neck Surgery, University Medical Center Utrecht, Utrecht, Netherlands; ^3^University Medical Center (UMC) Utrecht Brain Center, Utrecht, Netherlands; ^4^Department of Otorhinolaryngology, Donders Institute for Brain, Cognition, and Behaviour, Radboud University Medical Centre Nijmegen, Nijmegen, Netherlands; ^5^Department of Biophysics, Donders Institute for Brain, Cognition, and Behaviour, Radboud University Nijmegen, Nijmegen, Netherlands

**Keywords:** bone-conduction device, binaural hearing, transcutaneous, unilateral atresia, children

## Introduction

Unilateral congenital atresia, a specific type of unilateral conductive hearing loss (UCHL) occurs in 1 in 1,500 births (1). Unilateral congenital atresia, a specific type of UCHL occurs in 1 in 10–20,000 births (2). It is characterized by a moderate to severe unilateral conductive hearing loss as a consequence of the absence of the ear canal. It is widely echoed that children born with congenital unilateral aural atresia (UAA) face multiple challenges, ranging from esthetic to communication challenges. However, the literature demonstrating problems related to unilateral hearing is limited and mainly presented in relation to unilateral sensorineural hearing loss (3–7). Traditionally many clinicians and researchers believed that one normal ear was enough and there was no need to intervene. Several studies demonstrated the contrary [e.g., (8)], non-etheless, there still seems to be an uncertainty in the clinical management of children with UHL. For teachers, it is not always easy to understand the limitations of listening with one ear since communication may work well in quiet conditions, whereas noisy classrooms may put a large challenge on children with UHL. Timely evaluation by parents, school/day-care and clinicians might help to effectively deal with these problems.

Localization of sounds and understanding of speech in complex listening situations are reported to be impaired (9). Grammar and school problems are more often seen than in normal hearing children (5, 6, 10). For children with UAA, surgical repair does not always lead to sufficient hearing improvement (11), and revision surgery is common (12–14). Bone-conduction devices (BCDs), especially the more powerful BCDs, have been proposed as standard care for patients with UCHL, when surgical repair is unfeasible, and a conventional hearing aid is not possible (15, 16). Initially, BCDs were only provided to children with bilateral conductive hearing loss (9, 17–19). However, because of their success, implantable BCDs have also been provided to children with UCHL, aiming to provide/restore binaural hearing (20, 21). However, the treatment is reported to be controversial and the variation in performance after treatment with a BCD remains unexplained (14, 22). The present paper focuses on establishing the care for children with UAA who have a contraindication for a conventional hearing aid because of the absence of an ear canal. Treatment options are non-invasive BCDs (23), the percutaneous coupled BCD, the Bonebridge (24, 25), The Vibrant Soundbridge (26), and recently the Osia® (27–30).

Application of a hearing implant for children with congenital UAA and for patients with acquired aural atresia has proven to be effective (31, 32). However, in the aided condition hearing is still limited compared to normal hearing children, especially in complex listening situation, possibly because implantation does not result in true binaural hearing (33–36).

## Scope

This document does contain the following sections:

- Causes for limited benefit of a BCD for congenital UAA.
- Non-use.
- Is age at intervention important for consistent use of BCD in UCHL?
- Non-invasive bone-conduction hearing devices.
- What to advice parents of children with UAA?
- A multidisciplinary approach.

## Causes for Limited Benefit of a BCD for Congenital Unilateral Aural Atresia

Several differences between bone-conduction hearing and hearing with a normal-hearing ear exist. These differences might put constraints on adequate binaural processing. BCDs are limited in their dynamic range, which is typically <40 dB, whereas it is ~110 dB for the normal hearing ear. Furthermore, BCDs and VSB have a limited bandwidth (500 Hz−4 kHz), and a digital processing time of 3–14 ms. Therefore, BCDs provide less valuable and precise auditory information compared to the contralateral normal hearing ear. In addition, because bone-conduction sounds are effectively propagated by the skull bone, interaction of sounds might occur in the normal hearing cochlea between the air-bone signal and cross-stimulation caused by the BCD (37). Results from a cadaver study using bilateral bone conduction stimulation show complex intracochlear pressure patterns as a result of the interactions of signals presented from the left and right (38). Therefore, directional hearing might be affected when combining a signal from a BCD with a contralateral air-borne signal, and the input from the two ears may not fuse properly (i.e., inaccurate binaural hearing). This results in omitted advantages of binaural hearing, especially in complex listening situations with multiple, spatially separated sound-sources (39–42), and in a loss of accuracy in localization of sounds (43, 44).

A solid view on which children with UCHL benefit from interventions is needed (45, 46). Objective tests demonstrate that some children with UCHL improve their directional hearing while others hardly benefit or even deteriorate (9, 33, 47). A possible explanation is that some children with UCHL rely on the monaural spectral and level cues provided by the normal hearing ear (their coping strategy). In other words, they might localize sounds when listening with one ear. A recent study confirmed that hypothesis and suggested that the BCD did not enable real binaural hearing for children (34). Similar outcomes were found when using a device where cross-stimulation does not occur (viz. the Vibrant-soundbridge middle ear implant), suggesting that cross stimulation is not the main limiting factor for benefit (33, 48).

## Non-use

Research into the effectiveness of amplification in children with congenital UCHL is scarce and there is limited information about the long-term use of BCDs. A long-term evaluation by Nelissen et al. (49) demonstrated that almost half of the children with congenital UCHL discontinued use of their BCD within a few years (<5 year) after implantation. According to subjective reports from the children, the benefit of the device was limited, the device was disturbing in noisy situations and children turn down the volume of their BCD. Furthermore, some children simply didn't like to wear the BCD because of stigmatism, their disability becomes visible.

It would be of high prognostic value to understand the reasons for the high proportion on non-use. Is gain/sound itself annoying? Is it the delay between the ears? Might there be a chance that we implant too late? Would non-use decrease if frequencies around 6–8 kHz are better amplified?

## Is Age at Intervention Important for Consistent Use of BCD in UCHL?

We face several unanswered questions when dealing with congenital UCHL. One of the most important questions is whether there is an optimal window for the treatment of congenital UCHL. Recent work by Vogt et al. (34) demonstrates that implantation around 4–6 years does not result in better performance compared to implantation between 6 and 10 years. However, the results are not generalizable (i.e., based on a relatively small data set), and future research in children with UCHL receiving amplification through a BCD before 4 years of age is warranted.

Children with congenital UCHL can still hear their own voice by bone- and tissue conduction. This input (own voice) might minimize detrimental effects of a “sensitive period” and might explain why relatively early implantation (at age 4 years) has no advantage compared to implantation later in life (34). Yet, animal (50) as well as human (51) studies show that also in conductive hearing losses with normal inner ear function, cochlear synaptopathy can occur (50, 51). It is thus not yet clear if there is a limited time window in which implantation has to take place. Considering implantation in children, there is no scientific evidence that early implantation provides an advantage over implantation later in life (34). However, if clinicians wait till children can make their own decision, suboptimal organization of the auditory system because on unilateral innervation might have occurred already. Still, patients, parents and clinicians might consider waiting to implant children until they can make the decision regarding implantation themselves. If parents choose not to provide technical treatment, it is important to provide clear information to them, relatives and teachers, regarding possibilities and challenges when listening with one ear. Furthermore, it is important to provide information on how they can limit the functional deficits of listening with one ear. Finally, it is essential to monitor the development of the child, especially the speech and language development, and start therapy in time, because children with one ear are at risk (52, 53).

## Non-invasive Bone-Conduction Hearing Devices

Although more limited in technical performance compared to implantable BCDs [e.g., (54)], non-invasive (conventional) transcutaneous BCDs are also available. Non-invasive transcutaneous devices that are currently on the market are: a Baha/Ponto device on softband or headband (Baha®, Cochlear and Ponto® Oticon), the Baha® on a SoundArc®, the Contact MINI®, and the adhesive BCD (Adhear®, Medel). Non-invasive BCDs are provided to children with congenital UCHL for two reasons. Firstly, to provide access to sounds in the period prior to surgical placement of the more powerful percutaneous BCD. Secondly, to help to decide regarding implantation of a BCD. Early stimulation of the cochlea of the impaired ear provides bilateral input, which might enable proper cortical organization, and possibly reduce the risk of cochlear synaptopathy (51). It has been reported that listening entirely monaurally can result in massive reorganization of the auditory system (55). Ideally, bilateral input prior to implantation results in adequate maturation of the auditory system. It is, however, demonstrated that reweighting of the cues used for sound localization as well as adjustment of binaural sensitivity occurs for UHL, such that the imbalance caused by the UHL is offset (56, 57). If such reweighting has occurred, providing amplification may not give immediate benefits.

## What to Advise Parents of Children With UAA?

Hopefully the development of new hearing implants for conductive hearing loss (28–30, 58, 59) results in an increased compliance compared to percutaneous BCDs. These new devices might provide an alternative for the current suboptimal generation of BCDs although both the functionality and the increased costs for this system need to be considered (60). Implantation of the new active transcutaneous devices is more invasive compared to the conventional percutaneous BCDs. The implants contain titanium bodies and require a more complex surgery. Because of the complexity regarding possible treatment a multidisciplinary approach is needed to further improve support for children with UCHL. The ear implanted with a BCD, Bonebridge or Vibrant Soundbridge still cannot compete with the normal hearing ear. Remaining asymmetric hearing, processing delays, transcranial transmission, and a limited bandwidth in amplified frequencies might cause the suboptimal hearing abilities. Moreover, the ability of BCDs to provide true binaural hearing is limited. BCDs do not allow for processing of interaural time differences (33, 34), which is an important strategy for sound localization and hearing in background noise. Professionals might consider to advise parents of children with UCHL to wait with a more invasive hearing treatment until the children can make the decision regarding treatment themselves (52). Information for children, parents and teachers is important to further improve the care for these specific group of patients. In conclusion, because there are still many questions regarding the optimal treatment of this population patients with UCHL should not automatically be treated with a BCD.

## A Multidisciplinary Approach

Aiming for optimal care might seem trivial but we have to accept that the industry aims to sell as many implants as possible, that a more complex surgery suggests a better outcome and that the audiologist is aiming for the best possible aided thresholds. In order to realize optimal treatment, adequate information about the limitations and possibilities about monaural hearing should be provided by a multidisciplinary team. In order to limit overuse of medical treatment (61), this team should at least contain health-care professionals with a different background, for example an audiologist, ENT-surgeon and psychologist.

## Author Contributions

All authors contributed to the writing of the manuscript and all authors were involved in defining the topic.

## Conflict of Interest

The authors declare that the research was conducted in the absence of any commercial or financial relationships that could be construed as a potential conflict of interest.

## Abbreviations

BCD: bone-conduction device
UAA: unilateral aural atresia
UCHL: unilateral conductive hearing loss.
